# Intravascular lymphoma forming massive aortic tumors complicated with sarcoidosis and focal segmental glomerulosclerosis: a case report and literature review

**DOI:** 10.1186/s12882-018-1106-z

**Published:** 2018-10-29

**Authors:** Yasuhiro Oda, Kunihiro Ishioka, Takayasu Ohtake, Shuku Sato, Yotaro Tamai, Rikako Oki, Kenji Matsui, Yasuhiro Mochida, Hidekazu Moriya, Sumi Hidaka, Shuzo Kobayashi

**Affiliations:** 1 Department of Kidney Disease and Transplant Center, Shonan Kamakura General Hospital, 1370-1 Okamoto, Kamakura, Kanagawa 247-8533 Japan; 2 Department of Hematology, Shonan Kamakura General Hospital, 1370-1 Okamoto, Kamakura, Kanagawa 247-8533 Japan

**Keywords:** Intravascular lymphoma, Diffuse large B-cell lymphoma, Aortic tumor, Sarcoidosis, Focal segmental glomerulosclerosis, Adhesion molecule, Atherosclerosis, T-cell abnormality

## Abstract

**Background:**

Intravascular large B-cell lymphoma (IVLBCL) is a rare subtype of extranodal diffuse large B-cell lymphoma characterized by proliferation of B cells within small vessels. Herein, we report a case of a 77-year-old man who presented with IVLBCL and massive tumor formation on the aortic wall who was previously diagnosed with sarcoidosis and focal segmental glomerulosclerosis (FSGS). To our knowledge, this is the first reported case of an IVLBCL with aortic tumor formation.

**Case presentation:**

A 77-year-old ambulatory man with sarcoidosis and FSGS had neurological symptoms for nine months. The patient presented to the emergency department with sudden left leg pain, and was diagnosed with acute femoral artery occlusion. Emergency thrombectomy was performed subsequently. Pathological evaluation of the thrombi revealed that its surface was filled with large atypical B cells. Bone marrow biopsy showed infiltration of large atypical B cells within the small vessels. IVLBCL was suspected and further examination was planned, but the patient died due to sudden respiratory and cardiac arrest on hospital day twelve. Autopsy revealed intravascular tumors adherent to the aortic arch, left ventricle, and the abdominal aorta. All enlarged lymph nodes and the ventricular septum of the heart showed hyalinized lesions with granular formation consistent with sarcoidosis. The patient was diagnosed with IVLBCL with aortic tumor formation complicated with sarcoidosis and FSGS.

**Conclusions:**

IVLBCL may present with tumor formation on the aortic wall. Although the cause of its affinity to the aortic wall is yet unknown, autopsy findings imply that arteriosclerosis may have contributed to the tumor formation. The literature suggests that T-cell abnormalities could possibly be the common etiology of intravascular lymphoma, sarcoidosis, and FSGS.

## Background

Intravascular large B-cell lymphoma (IVLBCL) is a rare subtype of extranodal diffuse large B-cell lymphoma (DLBCL) characterized by the selective growth of B cells within small vessels [1]. It initially presents with various symptoms, which hinder early diagnosis and contribute to its high mortality and high frequency of post-mortem diagnosis [2]. Case series have shown that European patients present cutaneous and/or neurological symptoms more often than Asian patients, while Asian patients present hemophagocytic syndrome more often than European patients [2, 3]. Although its diverse presentations have been investigated, no case of IVLBCL with aortic tumor formation has been reported to date. In this article, we report the case of a 77-year-old man with focal segmental glomerulosclerosis (FSGS) and sarcoidosis presented with IVLBCL with massive tumor formation on the aortic wall.

## Case presentation

We present the case of a 77-year-old ambulatory man with hypertension, sarcoidosis, complete atrioventricular block status post-pacemaker implantation, chronic kidney disease due to FSGS, and right facial nerve paralysis, who presented with sporadic gait and right face numbness. He was diagnosed with sarcoidosis by biopsy of a tumor in front of the right tibia 14 years before presentation. Since the tumor and abdominal lymphadenopathy were the only manifestation of sarcoidosis and no other signs of organ involvement were present, he received no immunosuppressive treatment. The abdominal lymphadenopathy had been stable over time. Nine years before presentation, he was referred to our nephrology clinic to determine the cause of chronic kidney disease. His serum creatinine level was 1.2 mg/dL and he had proteinuria of 0.4 g per day. Hematuria was not present. Renal biopsy revealed six globally sclerotic glomeruli among all 34 glomeruli (18%) and some residual glomeruli with segmental sclerosing lesions, but no involvement of sarcoidosis. He was diagnosed with primary FSGS. Since the proteinuria was mild, he did not receive immunosuppressive treatment.

One year after that, the patient experienced palpitations and was diagnosed with complete atrioventricular block. Coronary angiography showed no significant stenosis of the coronary arteries, and he underwent pacemaker implantation. Whether sarcoidosis contributed to the complete atrioventricular block was unclear. The abdominal lymphadenopathy and the dyskinesia of the ventricular septum were stable and did not progress over time.

The patient was stable for eight years, until when he started to suffer from sporadic gait and right face numbness that occurred and resolved within a day every few weeks. Three months later, the symptoms recurred along with sudden dysarthria and left limbs weakness. Physical findings were notable for pronator drift on the left side. Perfusion computed tomography (CT) with iodinated contrast and CT angiography revealed no ischemic lesions or occlusion of major cerebral arteries. The symptoms disappeared three hours after the onset. A transient ischemic attack (TIA) was suspected, and he was admitted to the stroke unit. Ultrasonography revealed no stenosis of the internal carotid arteries, and transesophageal echocardiogram showed no abnormalities of the atrial septum. His pacemaker detected paroxysmal atrial fibrillation, which was presumed to be the etiology of the TIA. Thus, edoxaban 30 mg per day was started and he was discharged after one week of hospitalization.

One month after his discharge, his left leg started to swell and his gait worsened. Urinary protein excretion was 0.6 g per day, serum creatinine was at the baseline level of 1.6 mg/dL, and serum albumin level was 3.8 g/dL. Although no coagulopathy was found, ultrasonography revealed left femoral vein thrombosis that was 41 mm long. Edoxaban was stopped, and heparin was administered intravenously for two weeks. Low mobility due to his gait was presumed to be the cause of development of deep vein thrombosis (DVT). The patient was switched to warfarin and was discharged, but the left leg edema persisted. Three months later, he developed complications of urinary retention and constipation.

**Fig. 1:**
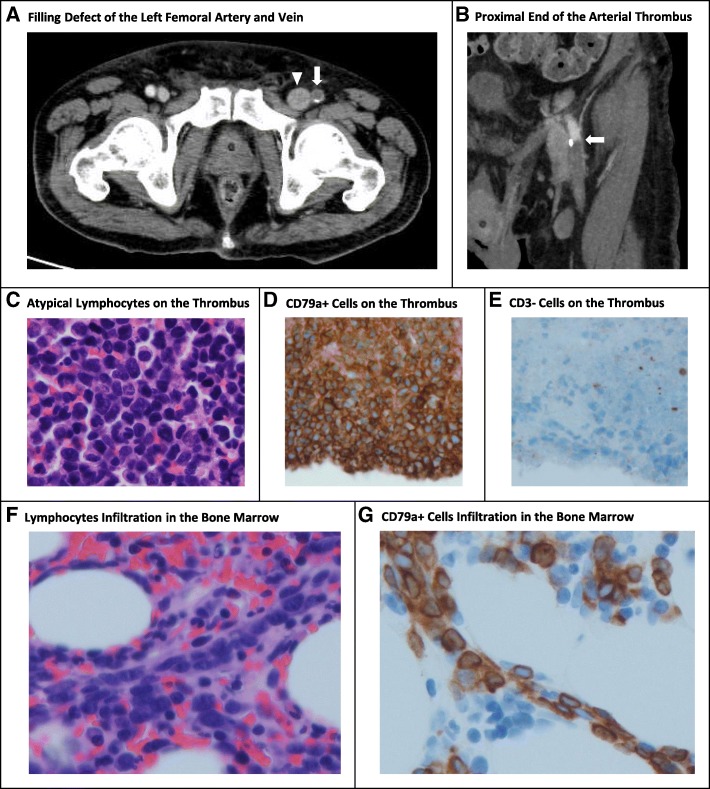
Radiologic, microscopic and immunohistochemical findings of the arterial thrombus and the bone marrow. CT scan with contrast showed filling defect of both femoral artery (Panel **a**, arrow) and dilated femoral vein (Panel **a**, arrowhead). Coronal image shows the proximal end of the thrombus in the left femoral artery (Panel **b**, arrow). Microscopic evaluation of the left femoral artery thrombus revealed fibrinoid necrotic lesion in the interior surrounded by large atypical lymphoid cells on the surface (Panel **c**; staining with hematoxylin and eosin (H&E)). Lymphoid cells were positive for CD20 and CD79a (Panel **d**) but were negative for CD3 (Panel **e**), which is characteristic to B cells. Bone marrow specimen revealed infiltration of large atypical lymphoid cells within small vessels (Panel **f**; H&E). Lymphoid cells within the small vessels were positive for CD79a (Panel **g**)

Four months after discharge, the patient presented to the emergency department with sudden left leg pain and inability to walk. The entire left lower limb was slightly pale and had slow pitting edema. The left dorsal artery was not palpable, and the left femoral artery was barely palpable. Contrast CT revealed occlusion of the left femoral and superficial femoral arteries together with the known DVT in the left femoral vein (Fig. 1a, b). Emergency thrombectomy for acute arterial occlusion was performed and the leg perfusion resumed. The emboli (maximum of 23 mm in diameter) were sent for pathological examination. The patient was admitted to the hospital and started on heparin infusion in place of oral warfarin. The history of recent TIA implied hypercoagulable state, but again no coagulopathy was found. While malignancy screening was being planned, the pathology of the arterial emboli revealed an unusual and surprising finding: the surface of the thrombi was filled with large atypical lymphoid cells (Fig. 1c) and was covering the necrotic interior of the thrombi. Immunohistochemical analysis showed that the tumor cells on the surface and the necrotic interior of the thrombi were positive for CD20 and CD79a but negative for CD3 (Fig. 1d, e), which is characteristic of B cells. Leukocytosis was absent (white blood cell, 4,000/μL; segmented neutrophil, 55%; lymphocyte, 34%; monocyte, 9%; eosinophil, 2%). Serum soluble interleukin-2 receptor level was 1,548 U/mL (normal, 122–496 U/mL); lactate dehydrogenase (LDH) level, 808 U/L (normal, 120–245 U/L); LDH-2 fraction, 39% (normal, 28–35%), and LDH-3 fraction, 32% (normal, 21–27%). These findings were consistent with large B-cell lymphoma with intravascular proliferation, but the etiology of the aortic thrombi was unclear.

The hematology consultation team considered that the patient needed further biopsy to determine the etiology. Bone marrow biopsy showed normocellular marrow with normal maturation, but with infiltration of CD79a-positive large atypical lymphoid cells within the small vessels (Fig. 1f, g). Although no lymphadenopathy was detected on palpation, CT scan showed swollen bilateral axillary and inguinal lymph nodes, which were up to 30 mm in diameter. While surgical biopsy of the right axillary lymph node and random skin biopsy were planned for diagnosis, the patient developed a complication of sepsis presumably due to pyelonephritis on hospital day nine. Piperacillin/tazobactam and vancomycin were started. Because partial thromboplastin time was prolonged, biopsies were withheld. Although white blood cell and neutrophil counts were improving, the patient died due to sudden respiratory and cardiac arrest on hospital day twelve. The patient had a do-not-resuscitate order. His family agreed to an autopsy.

## Pathological findings

An autopsy was performed consequently. In addition to gross and microscopic evaluations, immunochemical studies were performed to identify the immunophenotype of the cells in the lymph nodes, skin, intravascular thrombi (tumors), aorta, and lastly, medium and small vessels in and around the organs.

### Aortic tumor

A light brown-colored tumor, which was 40 mm in diameter, was macroscopically found at the cardiac side of the aortic arch. The tumor was adherent to the aortic wall and did not come apart by gently drawing it against the aorta. A 7-mm tumor with a similar color was found just above the origin of the superior mesenteric artery and a 15-mm tumor just below the left renal artery. There were also several flat brown-colored tumors adherent to the abdominal aorta. The aorta was also notable for atherosclerotic lesions.

A rectangular tumor, light beige in color and measuring 53 mm long and 13 mm wide, was found in the left ventricle and was firmly adherent to the trabeculae carneae. It was not obstructing the arterial valve or the mitral valve. No thrombus was found in the coronary arteries, pulmonary arteries, or veins.

**Fig. 2:**
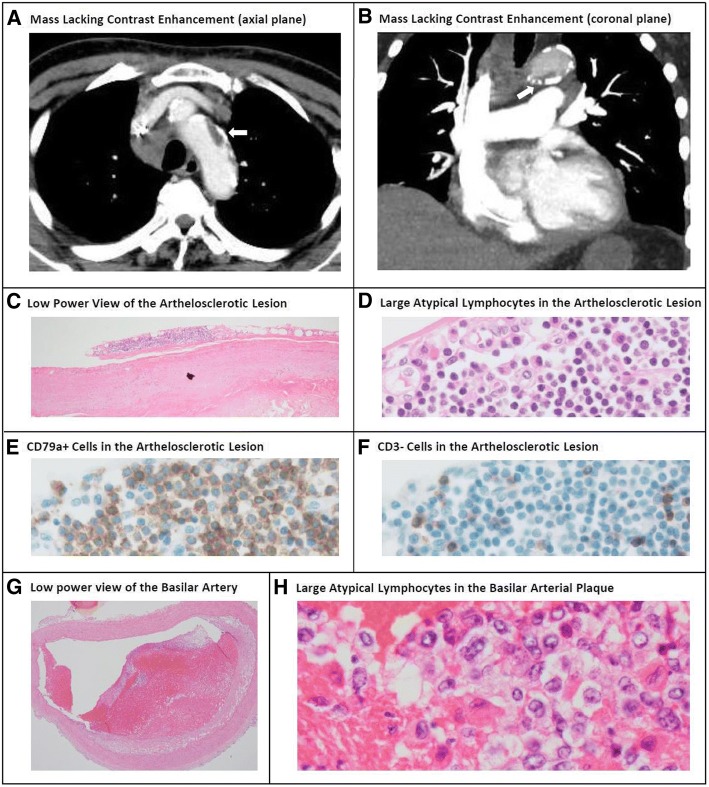
Radiologic, microscopic and immunohistochemical findings of the aortic tumor, artherosclerotic lesion, and basilar artery. CT scan with contrast showed a mass lacking contrast enhancement on the aortic arch (Panel **a** and **b**, arrows). Randomly selected artherosclerotic lesions of the aorta with no visible tumor also contained atypical lymphoid cells on its surface (Panel **c** and **d**; staining with H&E). These lymphoid cells were positive for CD79a (Panel **e**) and negative for CD3 (Panel **f**). Aggregated atypical lymphoid cells were also found in a plaque of the basilar artery (Panel **g** and **h**; H&E)

Tumors on the aortic arch and in the left ventricle were resected and subjected to microscopic evaluation. Both tumors contained aggregated atypical large lymphoid cells. Immunohistochemical analysis revealed that the tumor cells were CD79a positive and CD3 negative, which is consistent with the profile of the lymphoid cells in the femoral arterial thrombi. Reviewing the CT images retrospectively, a mass lacking contrast enhancement on the aortic arch, which was initially thought to be a chronic aortic dissection or artherosclerotic lesion, turned out to be the aortic tumor of the lymphoma (Fig. 2a, b).

Specimens from randomly selected artherosclerotic lesions of the aorta were also evaluated microscopically. Some, but not all, of these lesions contained aggregated atypical large lymphoid cells (Fig. 2c, d) that were positive for CD79a (Fig. 2e) and negative for CD3 (Fig. 2f).

### Intravascular lymphoma

Aggregation of atypical lymphocytes was found in the small vessels in the subcutaneous tissue but not in any of the lymph nodes evaluated microscopically. Specimens were obtained randomly from the kidneys and lungs to investigate small vessel involvement of the lymphoma, but no specimen contained atypical lymphocyte infiltration. Kidney specimens showed no major changes from those obtained nine years before.

The brain and its surrounding vessels were evaluated. The cerebrum, cerebellum, midbrain, pons, and medulla oblongata appeared normal on macroscopic and microscopic evaluation with no apparent infarction, hemorrhage, or infiltration of atypical lymphoid cells. However, aggregations of atypical lymphoid cells were found within the plaque of the basilar artery (Fig. 2g, h) and the vessels surrounding the midbrain and the medulla oblongata. The tumor cells were positive for CD79a and negative for CD3.

### Sarcoidosis

**Fig. 3:**
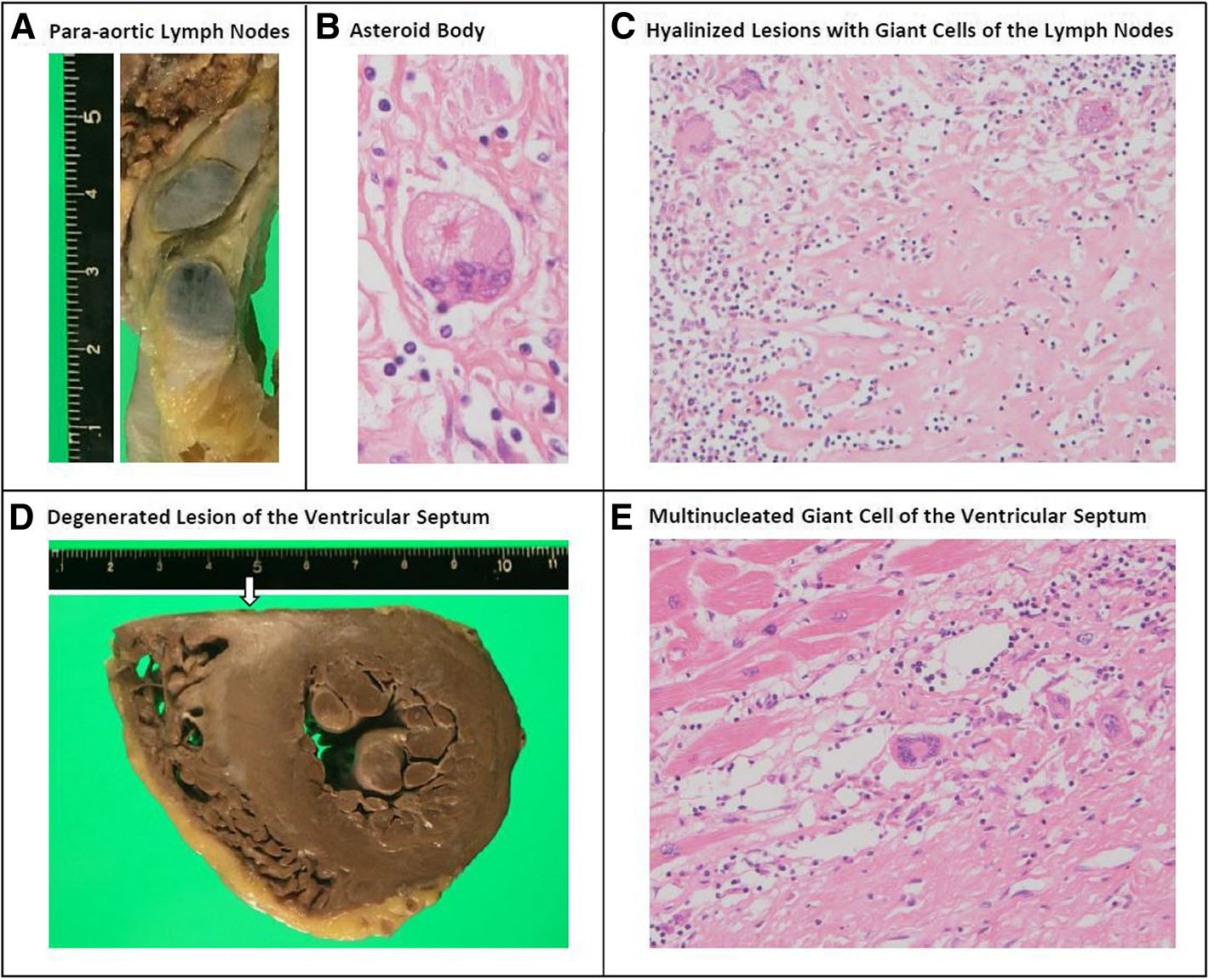
Macroscopic and microscopic findings of the abdominal para-aortic lymph nodes and left ventricle. Abdominal para-aortic lymph nodes were enlarged (Panel **a**; scale in centimeters). Their microscopic analysis revealed asteroid body and hyalinized lesions with multinucleated giant cells (Panel **b** and **c**; staining with H&E). Ventricular septum had white degenerated lesion seen macroscopically (Panel **d**, arrow; scale in centimeters), which contained multinucleated giant cells seen microscopically (Panel **e**; H&E). These findings are consistent with sarcoidosis with cardiac involvement

The cervical, mediastinal, hilar, para-aortic and mesenteric lymph nodes were all enlarged (Fig. 3a). Microscopic analysis revealed no aggregation of atypical lymphocytes but showed hyalinized lesions with granuloma formation and multinucleated giant cells (Fig. 3b), which were consistent with sarcoidosis. On macroscopic evaluation of the heart, the ventricular septum showed white fibrosis-like lesion. Microscopic analysis revealed multinucleated giant cells surrounded by hyalinized lesions, which is consistent with cardiac sarcoidosis.

## Discussion

IVLBCL is an uncommon subtype of extranodal DLBCL that is characterized by large B-cell proliferation in the lumina of small vessels [1]. However, no case of IVLBCL with aortic tumor formation has been reported to date. To our knowledge, this is the first reported case of an IVLBCL with aortic tumor formation.

Neurological symptoms with or without radiologic findings of stroke are known to be one of the primary symptoms of IVLBCL, particularly in Western-type IVLBCL [2, 3]. This case suggests that acute arterial occlusion may also be a presenting symptom of IVLBCL. Although the DVT of the left femoral vein was not evaluated pathologically, it could also have been the tumor of the lymphoma considering the odd fact that the DVT did not resolve with four months of anticoagulation therapy. Although the cause of death was unclear from the autopsy results, the history of sudden respiratory and cardiac arrest and the prominent tumor formation in the aorta imply that vascular occlusive events may have caused the patient’s death.

Whether sarcoidosis and FSGS had a role in IVLBCL development or in aortic tumor formation is of an academic interest. A large retrospective cohort study of patients with sarcoidosis in Sweden found that the risk of complicating non-Hodgkin’s lymphoma was high throughout the follow-up period [4]. Miyara et al. suggested a hypothesis that sarcoidosis is associated with a global amplification of regulatory T cell subset [5]. This might enhance the proliferation of naïve and effector T cells that produce interleukin-2, which overly acts as a growth factor for B cells and cause their transformation into malignant B cells [6]. This hypothesis may explain why B-cell malignancies develop several years after the onset of sarcoidosis. To our knowledge, this is the first report demonstrating a definitive case of sarcoidosis complicated with IVLBCL.

It is also of note that B cells may be a common key player in the pathogenesis of both sarcoidosis and FSGS. Recent research has shown that patients with sarcoidosis have altered B cell subsets in the peripheral blood [7, 8] and the altered subsets may reflect the disease activity of sarcoidosis [8]. Increasing number of case reports demonstrate that rituximab, an anti-CD20 monoclonal antibody, was effective in treating refractory sarcoidosis [9, 10] and refractory FSGS [11, 12]. There are several case reports on sarcoidosis complicated by FSGS [13, 14], but whether T-cell and/or B-cell function abnormality is the common underlying etiology is still under debate [15].

The mechanism of lymphoid cells adherence to the aortic wall is unknown. One study reported that tumors of primary central nervous system malignant lymphomas (PCNSMLs) tended to be positive for intercellular adhesion molecule-1, integrin-β1, matrix metalloproteinases (MMP)-2, and MMP-9, while none of these were expressed in the cells of intravascular lymphomas with central nervous system infiltration [16]. These molecules are known to mediate leukocyte adhesion or invasion [17], and were suggested to contribute to the invasive character of PCNSMLs. A recent report showed that junctional adhesion molecule-A was highly expressed in DLBCL patients with multiple extranodal lesions, and its overexpression may trigger transforming growth factor-β/NODAL signaling, cause B-lymphoma cell aggressiveness, and promote extranodal involvement [18]. It is possible that certain adhesion molecules and/or gelatinases caused the aortic tumor formation in our present case.

Another hypothesis arises from an interesting autopsy finding: the aggregation of lymphoid cells was not only present in the macroscopic tumors on the aortic wall, but also present in the arteriosclerotic lesions without apparent visible tumor. The patient had a 40-year history of hypertension and was on antihypertensive drugs, a 30-year history of type two diabetes mellitus and was on oral hypoglycemic agents, and a 10-year history of chronic renal disease due to FSGS confirmed by renal biopsy, all of which conferred a high risk of developing artherosclerosis. The pathological findings suggest that the tumor cells may have had a component that has an affinity to artherosclerotic lesions or vice versa, which may have contributed to the tumor formation on the aortic wall.

Evaluating cases with similar presentation may help to further investigate the etiology of IVLBCL with aortic tumor formation. There have been case reports of intra-aortic B-cell lymphoma confirmed by pathological evaluation [19] or suspected from history [20, 21]. None of them were proven to have IVLBCL, but there may be a common underlying etiology that promotes intra-aortic tumor formation.

## Conclusion

IVLBCL may present with tumor formation on the aortic wall. Although its etiology is yet unknown, molecules associated with cell adhesion or arteriosclerosis of the aorta may be the contributory factors for aortic tumor formation of the lymphoma. Previous studies suggest that T-cell abnormalities could be the common etiology of intravascular lymphoma, sarcoidosis, and FSGS.

## Abbreviations

CT: Computed tomography
DLBCL: Diffuse large B-cell lymphoma
DVT: Deep vein thrombosis
FSGS: Focal segmental glomerulosclerosis
H&E: Hematoxylin and eosin
IVLBCL: Intravascular large B-cell lymphoma
LDH: Lactate dehydrogenase
MMP: Matrix metalloproteinases
PCNSMLs: Primary central nervous system malignant lymphomas
TIA: Transient ischemic attack
